# Bacterial community dynamics and associated genes in hydrocarbon contaminated soil during bioremediation using brewery spent grain

**DOI:** 10.1099/acmi.0.000519.v3

**Published:** 2023-06-20

**Authors:** Mabel Owupele Nnadi, Lewis Bingle, Keith Thomas

**Affiliations:** ^1^​ Faculty of Health Sciences & Wellbeing, University of Sunderland, Chester Road, Sunderland SR1 3SD, UK; ^2^​ Brewlab, Unit One, West Quay Court, Sunderland SR5 2TE, UK

**Keywords:** bacterial community profiling, *alkB*, *catA*, *xylE* catabolic genes, diesel metabolites, total petroleum hydrocarbons, brewery spent grain, bioremediation

## Abstract

Brewery spent grain (BSG) has previously been exploited in bioremediation. However, detailed knowledge of the associated bacterial community dynamics and changes in relevant metabolites and genes over time is limited. This study investigated the bioremediation of diesel contaminated soil amended with BSG. We observed complete degradation of three total petroleum hydrocarbon (TPH C10–C28) fractions in amended treatments as compared to one fraction in the unamended, natural attenuation treatments. The biodegradation rate constant (*k*) was higher in amended treatments (0.1021*k*) than in unamended (0.059*k*), and bacterial colony forming units increased significantly in amended treatments. The degradation compounds observed fitted into the elucidated diesel degradation pathways and quantitative PCR results showed that the gene copy numbers of all three associated degradation genes, *alkB*, *catA* and *xylE,* were significantly higher in amended treatments. High-throughput sequencing of 16S rRNA gene amplicons showed that amendment with BSG enriched autochthonous hydrocarbon degraders. Also, community shifts of the genera *Acinetobacter* and *Pseudomonas* correlated with the abundance of catabolic genes and degradation compounds observed. This study showed that these two genera are present in BSG and thus may be associated with the enhanced biodegradation observed in amended treatments. The results suggest that the combined evaluation of TPH, microbiological, metabolite and genetic analysis provides a useful holistic approach to assessing bioremediation.

## Data Summary

The DNA sequencing data generated and analysed during the current study are publicly available and have been deposited with NCBI Sequence Read Archive (SRA) with BioProject accession number PRJNA861128. https://www.ncbi.nlm.nih.gov/sra/PRJNA861128

**Impact Statement**

This report summarises findings relating to enhancing the bioremediation of diesel fuel using brewery spent grain as an amendment, and the associated bacterial populations, during incubation in an organic soil. The data provides information on the biodegradation of hydrocarbons, relates the presence of signature metabolites in the degradation pathway to specific hydrocarbon degradation genes and changes in the microbial population. The use of various amendments to enhance bioremediation has received increased interest in recent years and is recognised as having value in industrial practice. This is enhanced when routine amendment materials such as spent grain is used, rather than expensive waste treatments. Our data is novel in using the same treatment samples for a combination of biochemical analysis of hydrocarbons and their degradation metabolites with the genetic identification of genes and microbial communities to illuminate correlations at each time point during the degradation. Intrinsic BSG bacterial populations have also been identified and profiled.

## Introduction

Environmental contamination by petrogenic hydrocarbons is ubiquitous and diesel fuel is one of the most common pollutants of this type [1, 2]. It is introduced to the environment mainly by spillage during transportation and storage, thereby contaminating water and soil [2, 3]. The impacts of diesel pollution go beyond environmental degradation to include health risks for humans and other organisms due to the toxic, mutagenic and carcinogenic nature of the constituent hydrocarbons [4, 5]. Another concern is that hydrocarbon pollutants can accumulate in the high trophic levels of the food chain [6, 7].

Soil is a key natural resource that is essential to sustain life on Earth. Many key functions of soil are carried out by microorganisms, which also play important roles in various biogeochemical cycles [8, 9]. However, soil also acts as an ultimate repository for contaminants, accumulating contamination through precipitation and sedimentation from air and water. This contamination affects the soil microbiota, leading to a disruption of its functions [8, 10]. While physiochemical remediation methods such as landfilling and incineration are available, more environmentally safe, efficient and cost-effective remediation methods are sought to mitigate the impacts of soil diesel contamination [11].

Biodegradation is the natural attenuation of toxic contaminants by autochthonous microorganisms. However, the process is slow, and enhancement is needed to deal with high contaminant concentrations, the associated nutritional imbalances and toxicity to intrinsic microbial populations [12]. Thus, bioremediation techniques are employed under controlled conditions to enhance biodegradation. Bioremediation is an exploitation of the metabolic capabilities of microorganisms to transform contaminants into innocuous, mineralized products [13]. The efficiency of the biodegradation process is associated with the enzymatic potential of the resident microorganisms, enabling them to digest the various substrates in the relevant degradation pathways [14–16].

Hence, microbial populations are the agents of biodegradation and play a vital role in the decontamination of petrogenic hydrocarbons in soil [11, 17]. As such, analysis of the microbial community dynamics during bioremediation is essential to understand the response and adaptation of microbes to pollution and monitor the bioremediation process [18]. More specifically, analysis targeting the 16S rRNA gene as a molecular marker enables profiling of microbial communities and has been usefully applied to characterise microbial communities involved in bioremediation of various environments [3, 12, 19]. Monitoring the presence and abundance of hydrocarbon degradation genes in the degrading microbiome is also required to fully appreciate its catabolic potential. This can also provide useful biomarkers for estimating the bioremediation potential of contaminated sites [20, 21]. For example, the *alkB* gene is very important in the aerobic transformation of aliphatic hydrocarbons as it encodes an alkane monooxygenase enzyme that hydrolyses alkanes to their corresponding primary or secondary alcohols and has been evaluated in a number of studies [22–24].

Bacteria are instrumental in degrading hydrocarbon pollutants in soil and utilising the resulting metabolites for energy and growth, via the TCA cycle [25]. Metabolites associated with hydrocarbon degradation have also been successfully identified in studies [26, 27]. Following soil contamination, biodegradative bacterial strains that are resistant to the toxicity of soil pollutants can detoxify soil and make nutrients available for other quiescent populations to grow, leading to changes in microbial community structure and succession [28, 29]. As a result, community composition varies over time, with those species best able to exploit the metabolic breakdown products dominating as bioremediation progresses [30]. Bacterial genera associated with the biodegradation of petroleum hydrocarbons have been shown to include *Acinetobacter, Arthrobacter, Bacillus, Flavobacterium, Nocardia, Pseudomonas* and *Vibrio* [5, 31]. Among the different species associated with bioremediation, *Pseudomonas* spp. in particular are known to be versatile in the biodegradation of hydrocarbons, especially those found in diesel fuel [32]. However, knowledge of the microbial species involved in diesel remediation using food wastes in temperate countries is limited.

Shahsavari *et al*. [11] observed high degradation rates in diesel and gasoline contaminated soil amended with crop residues. As soil is the most expensive medium to decontaminate, the use of crop residues and food waste is cost-efficient and advantageous [33]. Brewery spent grain (BSG) is a readily available food by-product with high nutritional content [34, 35]. It has been successfully used to stimulate bioremediation and its effluents have been used for diesel bioremediation, indicating strong potential [36]. However, these uses have been mostly under tropical conditions [37, 38]. BSG was chosen as a biostimulant in this study because, as well as releasing nutrients for autochthonous bacteria during biodegradation, it is known to have its own resident microflora and so may provide both biostimulation and bioaugmentation benefits [39]. It would also allow reuse of an industrial by-product that might otherwise be put to landfill as waste [40].

Previous work on diesel biodegradation using BSG has tended to focus on the reduction of total petroleum hydrocarbon (TPH) and culturable bacterial count. Changes in the associated catabolic genes, metabolites and community succession over time during diesel bioremediation using BSG in a temperate soil have not been investigated. This time-course study was designed to illuminate any correlations between breakdown metabolites, gene abundance and community shifts in response to biodegradation, with the aims of evaluating the impact of BSG supplementation on diesel bioremediation in soil and answering the following questions. How does BSG influence biodegradation? What are the metabolites associated with this biodegradation process and how do they fit into known hydrocarbon breakdown pathways? Which bacterial species are associated with biodegradation, how does their abundance change over time and does BSG supplementation favour known hydrocarbon degrading bacteria and catabolic genes?

## Methods

### Soil preparation and experimental design

Soil was collected (10–30 cm depth) from an uncontaminated pristine agricultural farmland using sterile implements, air dried and sieved using a 4 mm mesh [11]. The pH of the soil was determined to be 6.8 after a 1 : 2.5 soil:distilled water dilution [41]. Soil characteristics were determined (by Anglian Soil Analysis) as listed in Table 1. The three treatments used in this study were soil+diesel (S+D) unamended natural attenuation, soil+diesel+BSG (S+D+G) amendment, and soil alone (S) control. In triplicate, 20 ml of diesel was spiked into 2000 g of soil to achieve a 1 % (v/w) contamination. To ensure homogeneity the diesel was added to 25 % of the soil and mixed thoroughly with a stainless-steel implement before adding the remaining soil [42]. BSG (10 %) was then added to treatments as required and mixed in thoroughly. BSG had a moisture content of 75% and a pH of 5.3 and was stored at 4 °C for 4 days prior to use.

**Table 1.** Physical and chemical characteristics of the soil used in this study

Soil property	Value
Soil texture	Sandy/Loam
pH	6.8
Moisture (%)	26.04
Organic matter (%)	12.0
Sand (%)	50.44
Silt (%)	41.84
Clay (%)	7.72
Total organic carbon (%)	6.96
Potassium (mg kg^−^¹)	15.4
Magnesium (mg kg^−^¹)	21.1
Phosphate (mg kg^−^¹)	17.5
Nitrate (mg kg^−^¹)	3.5

Sterile counterparts of treatments (sterile soil+diesel, and sterile soil+diesel+sterile BSG) were also analysed to confirm biotic loss. Sterilisation was achieved prior to diesel spiking by autoclaving for 1 h at 121 °C on three alternate days [43, 44]. For each treatment, 2000 g of soil was incubated in triplicate 5 litre pots. Treatment pots were covered with Gore-Tex cloth and incubated at 15±3 °C. Treatments were oxygenated by mixing twice a week using a sterile spatula and the moisture content was maintained by the weekly addition of 5 % (v/w) sterile distilled water. Composite samples for analysis were obtained from each pot on days 0, 2, 5, 7, 12, 14, 21 and 28 by collecting 5 g samples from the four corners and the centre and mixing together [45].

### Determination of TPH removal and detection of hydrocarbon metabolites

#### GC analysis

TPH was determined using a modified US EPA 8015 technique [46, 47]. Hexane was used for diesel extraction with mechanical shaking [23]. Aliquots of the extract (1 ml) were transferred in triplicate to 1.5 ml GC vials and GC analysis was carried out using an Agilent Technologies 7890A system equipped with a flame ionization detector (FID) and autosampler (7693). A 30 m×0.32 mm×0.25 µm capillary column (19091J-413E HP-5; Agilent Technologies) was used with helium as the carrier gas at a flow rate of 2 ml min^−1^, hydrogen gas at a flow rate of 30 ml min^−1^ and air at a flow rate of 300 ml min^−1^. The temperature programme used was a modified version of that given by Bento *et al*. [46]. The initial temperature was 50 °C with isothermal operation for 5 min, followed by heating to 270 °C at a constant rate of 10 °C min^−1^ and a final 5 min isothermal operation. Samples from each time point and triplicate standards were analysed on the same run.

#### Percentage TPH reduction, extent of aliphatic TPH (C10–C28) fractions removal and biodegradation rate

Percentage TPH reduction was calculated using the formula: %TPH reduction=[(TPH of control - TPH treatment)/TPH control]×100, with day 0 TPH being used as a control for each treatment [46]. The TPH Standard Mix 1 (Sigma Aldrich), with known concentration for each of the (C10–C28) analytes, was used to obtain the calibration curve for each fraction. Concentrations of each fraction in the soil hydrocarbon mixture were then determined based on the calibration curve of each corresponding standard fraction. Retention times of each fraction (analyte) in the standard were compared to the sample chromatogram to determine target compounds, and the total peak areas of both standard and analyte fractions were determined. The biodegradation rate was determined using the formula $\frac{C}{C_{0}}$ = $e^{{-k}^{t}}$ [48], which is same as: C = $C_{0}e^{{-k}^{t}}$ [37], where *C* is the concentration of the TPH fractions (mg kg^−1^) at time *t*, *C*_0_ is the initial concentration of the TPH fractions (mg kg^−1^), *t* is time (day^−1^) and *k* is the biodegradation rate constant (day^−1^).

#### GS-MS analysis

GC-MS analysis was carried out using an Agilent GC-MS 7890A/5975C series instrument with a 30 m×0.32 mm×0.25 µm capillary column (19 091S-433E HP-5MS; Agilent Technologies). Helium was the carrier gas with a flow velocity of 1 ml min^−1^ and 1 µl of sample was injected into the column in a splitless mode. The analytical conditions were an initial temperature of 50 °C, with isothermal operation for 1 min followed by heating to 120 °C at a constant rate of 20 °C min^−1^ and a final heating to 310 °C at a constant rate of 4 °C min^−1^ [49] with a 5 min isothermal operation. The column was directly connected to an electron ionisation mass spectrometer with an electron energy of 70 eV, producing ions that are characterised according to mass-to-charge ratio and relative abundance.

#### Detection of hydrocarbon degradation metabolites and BSG metabolic potential

Following the GC-MS analysis to identify compounds present in the treatments at each time point, the G3835AA Mass Hunter Mass Profiler Professional Software (Agilent Technologies) was used to analyse the MS data (identified compounds) and the identified metabolites were used to determine the oxidative pathways utilized in the breakdown of diesel with and without BSG. The abundance of each compound in the treatments, and compounds differing significantly between the treatments overall, were determined.

### Determination of colony forming units (CFUs) for the enumeration of heterotrophic and hydrocarbon-degrading bacteria

Composite samples of 10 g from each treatment pot were transferred to sterile bottles containing 100 ml of 0.2 % (v/v) sterilised sodium pyrophosphate and mixed on a shaker at 150 rpm for 30 min at 20 °C. Thereafter, 1 ml of the soil suspension from each bottle was 10-fold serially diluted in sterile saline to give dilutions 10^−1^ to 10^−6^. Following dilution, 0.1 ml of each suspension was plated onto R2A agar for enumeration of heterotrophic bacteria, and onto oil agar for enumeration of hydrocarbon-degrading bacteria. These media were incubated at 30 °C for 24 h and 25 °C for 7 days, respectively.

### Quantification of catabolic genes and bacterial community composition profiling

#### DNA extraction from treatments

Microbial community DNA was extracted from bioremediation treatment samples (1 g), with and without BSG at each time point (days 0, 5, 12 and 21), using the EZNA soil DNA kit (Omega Bio-Tek) [50]. The purity of extracted DNA was estimated by measuring absorbance at 260 and 280 nm, using a micro volume spectrophotometer (Nanodrop Technologies) and calculating the 260/280 ratio, which was required to be between 1.8 and 2.0.

#### qPCR quantification of diesel catabolic genes

Three hydrocarbon catabolic genes, *alkB* [51], *catA* and *xylE* [52], were assayed quantitatively using PCR primers as listed in Table 2. Quantification was performed by real-time quantitative PCR (qPCR) using 1 : 10 dilutions of the extracted community DNA in sterile nuclease-free water [52]. The assays were performed in a Rotor Gene Q thermocycler (Qiagen) using the 2× Kapa Sybr Fast qPCR Master Mix Universal kit (Sigma Aldrich). Each reaction (20 µl) contained 2× Kapa Sybr Fast qPCR Master Mix (10 µl), forward primer (0.2 µM), reverse primer (0.2 µM), PCR-grade water (8.2 µl) and DNA template (1 µl). The amplification programme for the *alkB* gene included initial denaturation at 95 °C (5 min), followed by 40 cycles of denaturation at 95 °C (10 s), annealing at 50 °C (30 s) and extension at 72 °C for 30 s, and primer–dimer removal and signal acquisition at 80 °C for 10 s. Thermal cycling programmes for both the *catA* and *xylE* genes comprised an initial denaturation step at 95 °C (5 min), followed by 40 cycles of denaturation at 95 °C (10 s), annealing at 58 °C (30 s) and extension at 72 °C (30 s), with primer–dimer removal and signal acquisition at 80 °C for 10 s. Reactions were run in triplicate and negative controls (PCR-grade water) were included in all amplifications.

**Table 2.** Details of the primers used for the detection and quantification of target hydrocarbon catabolic genes

Target gene	Primer name	Annealing temperature (°C)	Sequence (5′–3′)	Amplicon size (bp)	References
Alkane monoxygenase	*alkB*	50	F: AACTACATCGAGCACTACGG R: TGAAGATGTGGTTGCTGTTCC	100	[51]
Catechol-1,2-dioxygenase (C12O)	*catA*	58	F: ACVCCVCGHACCATYGAAGG R: CGSGTNGCAWANGCAAAGT	470	[81]
Catechol-2,3-dioxygenase (C23O)	*xylE*	58	F: AAGAGGCATGGGGGCGCACCGGTTCGATCA R: CCAGCAAACACCTCGTTGCGGTTGCC	380	[82]

In an initial experiment to evaluate the methodology [53], PCR amplicons from genes of interest in the amended treatment were verified to be of the correct band size by electrophoresis on a 2 % (w/v) agarose gel. Bands were visualized using a Chemi Doc MP Imaging System (Bio-Rad Laboratories), excised under UV radiation and extracted using the QIAquick Gel Extraction Kit (Qiagen). Following confirmation of their DNA sequences, the cleaned PCR products were used as positive controls for the standard curve. CT values of the treatments were related to the standard curve. Gene copy numbers were calculated using the formula: number of copies = (ng/µl DNA×6.022×10^23^)/(PCR product length in base pairs×1×10^9^×650) [54] where Avogadro’s number of 6.022×10^23^ is the number of molecules/mole DNA and 650 Da is the average weight of a base pair. Results were expressed as log_10_ of gene copy numbers per g dry soil (log_10_ g ^−1^).

#### High-throughput sequencing of 16S rRNA gene amplicons for bacterial community profiling

Samples of microbial community DNA extracted from bioremediation treatments with and without BSG at each time point (days 0, 5, 12 and 21), as used for catabolic gene quantification, were also used for community profiling via analysis of the 16S rRNA gene. V4 variable region PCR primers 515–806 [55] were used in a single-step 30-cycle PCR using the HotStarTaq Plus Master Mix Kit (Qiagen) under the following conditions: 94 °C (3 min), followed by 28 cycles (five cycles used on PCR products) of 94 °C (30 s), 53 °C for (40 s) and 72 °C (1 min), after which a final elongation step at 72 °C (5 mins) was performed.

Sequencing was performed at MR DNA using an Ion Torrent PGM system. Sequence data were processed using a proprietary analysis pipeline (MR DNA). In summary, sequences were depleted of barcodes and primers, then sequences <150 bp, with ambiguous base calls and with homopolymer runs exceeding 6 bp, were removed. Sequences were denoised, operational taxonomic units (OTUs) generated and chimaeras removed. OTUs were defined by clustering at 3 % divergence (97 % similarity). Final OTUs were taxonomically classified using blast against a curated database derived from Green Genes, RDPII and NCBI (www.ncbi.nlm.nih.gov, [56], http://rdp.cme.msu.edu). Data were submitted to the Sequence Read Archive (SRA) at NCBI with the BioProject accession number PRJNA861128 (https://www.ncbi.nlm.nih.gov/sra/PRJNA861128).

### Statistical analysis

IBM SPSS 24 statistics software was used to determine significant differences among treatments with respect to TPH reduction, microbial count and catabolic gene copy numbers. All data were tested for normality using the Shapiro–Wilk test. ANOVA and the least significant difference (LSD) post-hoc test were used to determine significant differences among treatment means of more than two independent variables, while the independent t test was used to determine significant differences between two independent variables. Significance was determined as *P*<0.05.

## Results

### Determination of TPH removal and detection of hydrocarbon metabolites

#### Percentage TPH reduction

The natural attenuation treatment (S+D) and the treatment amended with BSG (S+D+G) produced a rapid reduction in TPH of 78 and 84% respectively by day 2 (Table 3). Further reductions of 92 % in the natural attenuation and 96 % in the amended treatments were observed after 1 week of incubation. After 28 days of incubation, a final 99 % reduction was observed in the amended treatment and a 93 % reduction in the natural attenuation treatment. Statistical analysis revealed a significant difference in TPH total peak heights between the two treatments throughout the experiment except at day 0. The treatment amended with BSG showed a significantly higher percentage TPH reduction compared to the natural attenuation, unamended treatment.

**Table 3.** Percentage reduction of TPH during bioremediation of diesel-contaminated soil treatments with (S+D+G) and without (S+D) BSG

	TPH reduction in treatments (%)	
Day	S+D	S+D+G
2	78	84
5	89	92
7	92	96
12	92	97
14	92	97
21	93	98
28	93	99

#### Extent of aliphatic TPH (C10–C28) fraction removal and biodegradation rate

**Fig. 1.**
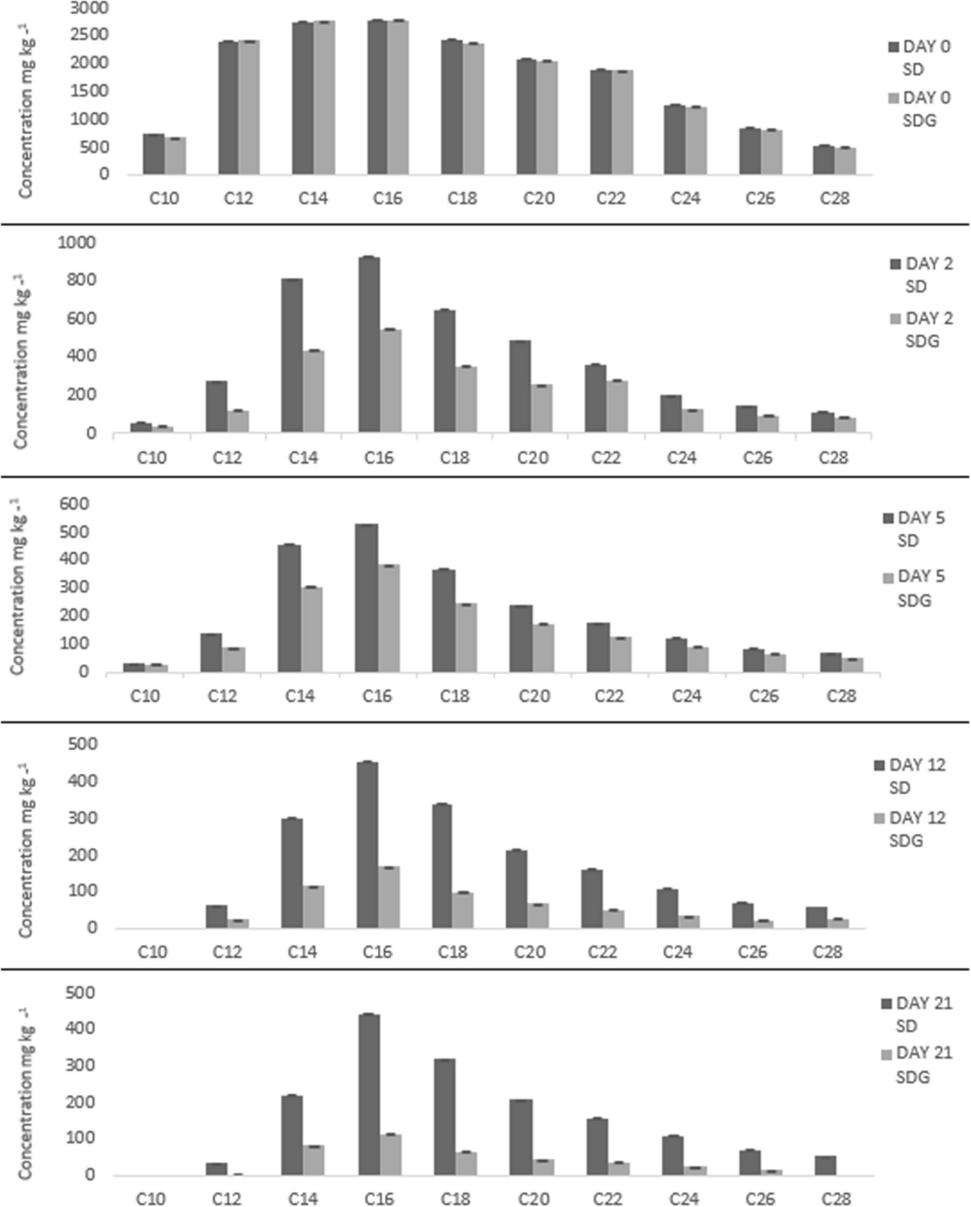
Extent of aliphatic TPH (C10–C28) fraction removal, with and without BSG, over time, during diesel biodegradation. Results represent the means of three replicates. Error bars show standard error. SD=soil+diesel, SDG=soil+diesel+BSG amendment.

The extent of aliphatic TPH fractions (C10–C28) removal in both natural attenuation (S+D) and BSG-amended treatments (S+D+G) was evidenced by the reduced concentrations of the TPH fractions over time during bioremediation (Fig. 1). The results show a 100 % decline of the C10 fraction by day 12 in both treatments. This was the only hydrocarbon fraction to be fully removed in the natural attenuation treatment. However, a complete decline of the C10, C12 and C28 fractions was observed in the amended treatment by day 28. These three fractions had initial concentrations of 700, 2400 and 500 mg kg^−1^ respectively at the start of the incubation. The concentrations of the C14 to C26 fractions reduced over time but persisted until day 21 in both treatments. However, the reduction of these fractions was significantly greater in the amended treatment than in the natural attenuation treatment. The biodegradation reaction rate constant (*k*) was significantly higher in the amended treatment (0.1021 day^−1^) compared to the natural attenuation treatment (0.0590 day^−1^). The first-order linear model *r* values were 0.8699 and 0.9585 respectively for these two treatments.

#### Detection of hydrocarbon degradation metabolites and BSG metabolic potential

Compounds detected by GC-MS from the treatments (S+D and S+D+G) at different stages during the bioremediation were assessed to determine the presence of known metabolites in the degradation pathways of both aliphatic and aromatic hydrocarbons, based on their functional groups such as alcohols, ketones, esters, aldehydes, carboxylic acids and esters.

Results for aliphatic hydrocarbon degradation (Table 4) revealed that ketones, being metabolites of the subterminal oxidation pathway, were present in both unamended (S+D) and amended (S+D+G) treatments, throughout the bioremediation process, while aldehydes, being metabolites of the terminal oxidation pathway, were absent. However, carboxylic acids, which are further metabolites of the terminal oxidation pathway, resulting from aldehyde oxidation [5, 57, 58], were present at the start and up until day 5 in the unamended treatment but were only present at the start in the amended treatment. For aromatic hydrocarbons, however, aromatic ketones, which are oxidation products of the *ortho*-cleavage pathway, were present in both treatments throughout the bioremediation. Aromatic aldehydes, which are oxidation products of the *meta*-cleavage pathway, were only observed from the start of the experiment on days 0 and 5 in unamended treatments but were present on days 5, 12 and 21 in the amended treatments.

**Table 4.** Hydrocarbon degradation metabolic compounds present in treatments, over time during bioremediation, including diesel control

				Treatment							
				Day 0		Day 5		Day 12		Day 21	
Hydrocarbon type	Functional group	Type of compound	Diesel control	Soil and diesel	Soil diesel and BSG	Soil and diesel	Soil diesel and BSG	Soil and diesel	Soil diesel and BSG	Soil and diesel	Soil diesel and BSG
**Aliphatic hydrocarbons**	C-H	STRAIGHT CHAIN	+	+	+	+	+	+	+	+	+
	–OH	ALCOHOLS		+	+	+	+	+	+	+	+
	–H–C=O	ALDEHYDES		−	−	−	−	−	−	−	−
	–R–C=O	KETONES		+	+	+	+	+	+	+	+
	–OH–C=O	CARBOXYLIC ACIDS		+	+	+	−	−	−	−	−
	–O–C=O	FATTY ACID ESTER		+	+	+	+	+	+	+	+
	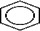										
**Aromatic hydrocarbons**		BENZENE RINGED	+	+	+	+	+	+	+	+	+
	–OH	CATECHOL (Alcohol)	+	+	+	+	+	+	+	+	+
	–H–C=O	ALDEHYDES		+	−	+	+	−	+	−	+
	–R–C=O	KETONES		+	+	+	+	+	+	+	+
	–OH–C=O	CARBOXYLIC ACIDS		+	+	+	+	+	+	−	−
	–O–C=O	FATTY ACID ESTER		+	+	+	+	+	+	+	+
											

### Determination of colony-forming units (CFUs) for the enumeration of heterotrophic and hydrocarbon-degrading bacteria

**Fig. 2.**
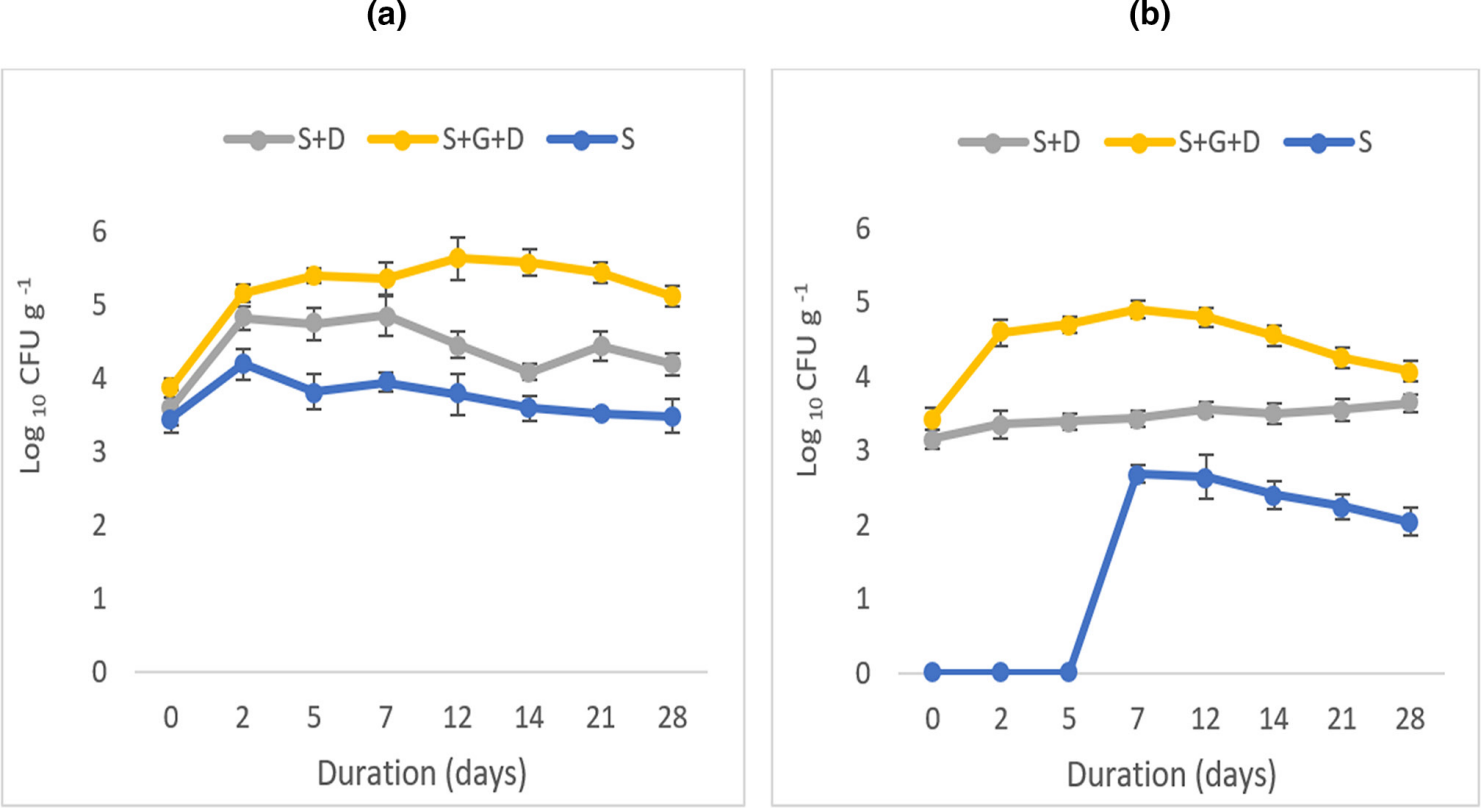
Mean colony-forming units (CFUs) for the enumeration of heterotrophic bacteria (**a**) and hydrocarbon-degrading bacteria (**b**) in treatments with and without BSG. Results represent the means of three replicates. Error bars show standard error. S+D=soil+diesel, S+D+G=soil+diesel+grain amendment, S=control soil.

Changes in heterotrophic bacterial CFUs are shown in Fig. 2a. CFUs in the unamended diesel contaminated soil samples (S+D) increased at the start of the experiment and peaked at 5.0 log_10_ g^−1^ on day 7 after which they decreased continually. CFUs in the diesel contaminated samples amended with BSG (S+D+G) increased continually from day 0 and peaked at 5.6 log_10_ g^−1^ on days 12 and 14 before decreasing gradually. However, the heterotrophic bacterial CFUs remained significantly higher in the amended treatment (S+D+G). The soil control treatment (S) also showed increased CFUs after day 0 but peaked at 4.2 log_10_ g^−1^ on day 2. This treatment had the lowest CFUs.

The soil control treatment (S) had no hydrocarbon utilising bacterial growth until day 7 after which the population slowly declined. Hydrocarbonoclastic CFUs in the unamended treatment (S+D) remained similar throughout the experiment (Fig. 2b), with a 6 % increase on day 12 when it peaked at 3.6 log_10_ g^−1^. The CFUs in the amended treatment (S+D+G) had the highest CFUs and showed a rapid increase of 34 % on day 2 as compared to the level at day 0. It then peaked at 4.9 log_10_ g^−1^ on day 7 with a 43 % increase compared to the unamended treatment (S+D). After this time, a gradual decrease in CFUs occurred until day 28 at which point a 12 % increase in CFUs was seen as compared to the unamended treatment. A negative correlation was observed between TPH concentration and hydrocarbon-degrading CFUs in both unamended (*r*=−0.858) and amended (*r*=−0.926) treatments.

### Quantification of diesel catabolic genes and bacterial community composition

#### Quantification of diesel catabolic genes by qPCR

**Fig. 3.**
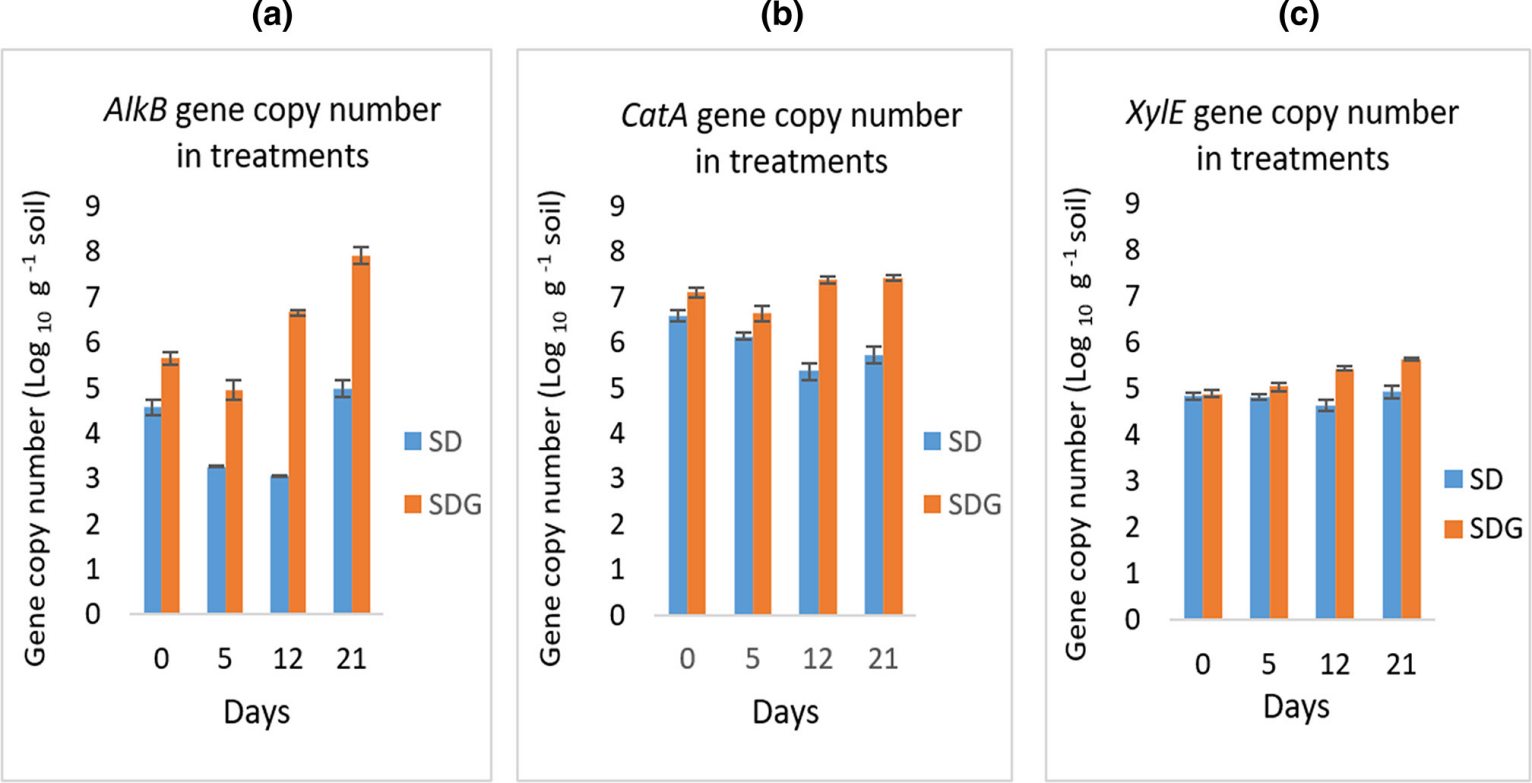
Gene copy numbers of the *alkB* gene (**a**), *catA* gene (**b**) and *xylE* gene (**c**) in treatments with and without BSG over time, during the bioremediation of diesel-contaminated soil. Results represent the means of three replicates. Error bars show standard error. S+D= soil+diesel, S+D+G=soil+diesel+grain amendment.

Gene copy number quantification in this study was limited to the contaminated soils, with and without BSG, to determine the bioremediation potential of BSG. The gene copy numbers of all three catabolic genes in this study increased with the addition of BSG. From day 5 until the end of the experiment, the *alkB* gene copy numbers (Fig. 3a) in the amended treatment (S+D+G) were significantly higher than that of the unamended treatment (S+D). Also, a very distinct difference in gene copy numbers was observed in the amended treatment on day 12 while that in the unamended treatment remained constant. Nevertheless, there was an increase in gene copy numbers in both treatments after this time. From the start to the end of the experiment, gene copy numbers in the unamended treatment (S+D) increased by 22.8 % from 4.57 to 5.61 log_10_ g^−1^ while that in the amended treatment (S+D+G) increased by 40.4 % from 5.65 to 7.93 log_10_ g^−1^.

A significant difference in the *catA* gene copy numbers was observed (Fig. 3b), between the amended (S+D+G) and unamended (S+D) treatments from the start and throughout the experiment. The rapid reduction in percentage TPH and concentration of aliphatic hydrocarbon (C10–C28) fractions also occurred at the start of the experiment and was associated with a negative correlation in hydrocarbon degrading bacterial CFUs. However, like the *alkB* gene, a distinct difference in gene copy numbers was evident between the amended and unamended treatments from day 12, after which it plateaued until the end of the experiment. Nevertheless, a 13.2 % decrease in the *catA* gene copy numbers, from 6.59 to 5.72 log_10_ g^−1^, was observed in the natural attenuation treatment (S+D) from day 0 to the end of the experiment. In the amended treatment (S+D+G), *catA* gene copy numbers increased by 4.7 % from 7.10 to 7.43 log_10_ g^−1^.

Gene copy numbers for the *xylE* gene (Fig. 3c), like the *alkB* gene, were significantly higher in the amended treatment from day 5 and throughout the experiment. However, as with both the *alkB* and *catA* genes, a distint difference in gene copy numbers was evident from day 12. A 1.9 % increase in copy number of the *xylE* gene was observed in the naturally amended treatment, (S+D), from day 0 to the end of the experiment increasing from 4.84 to 4.93 log_10_ g^−1^ while a 15.4 % increase was observed in the amended treatment, (S+D+G), from 4.89 to 5.64% log_10_ g^−1^.

#### High-throughput sequencing of 16S rRNA gene amplicons for community profiling

**Fig. 4.**
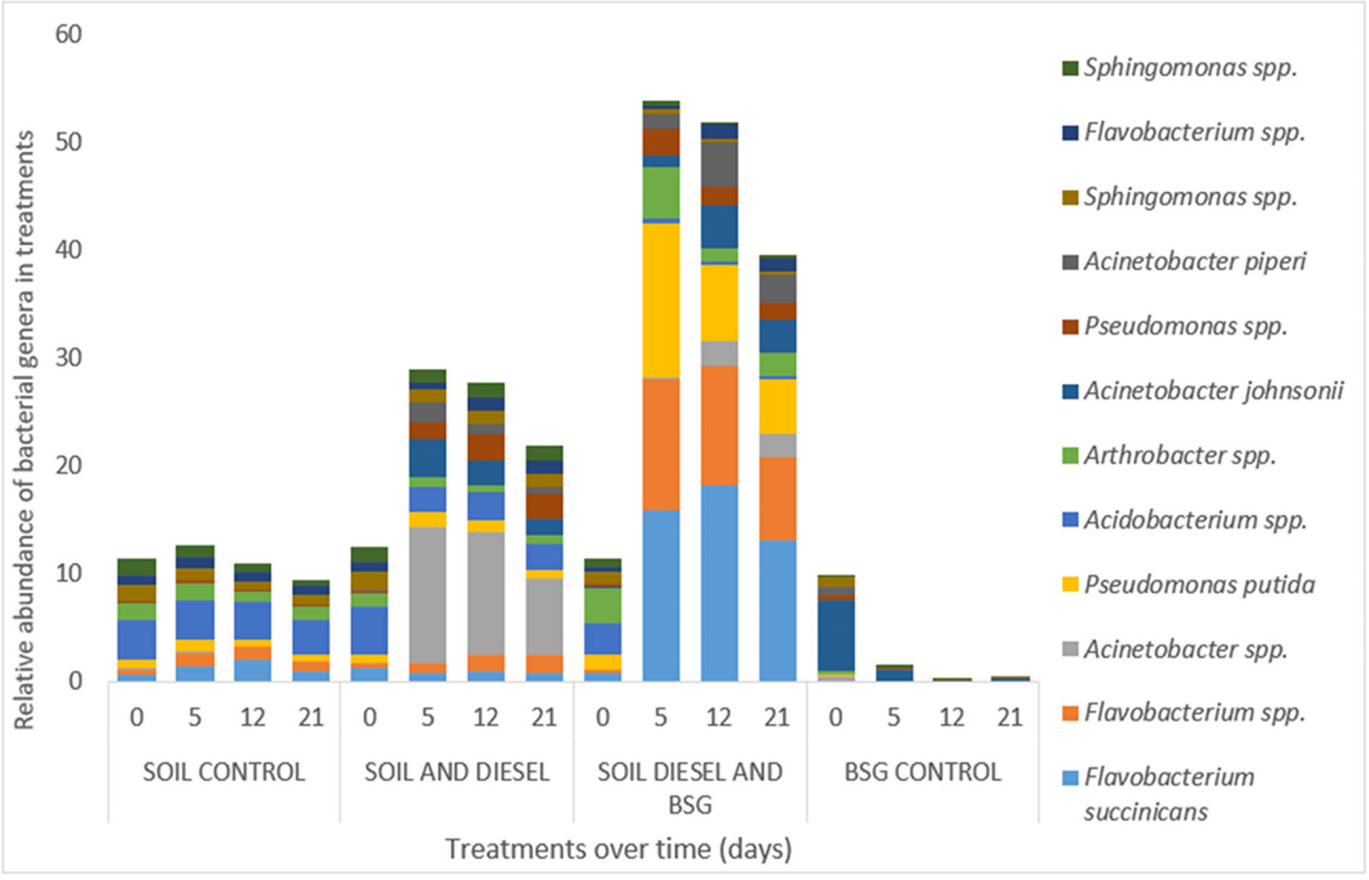
Relative abundance of bacterial genera present in samples showing the bacterial population profile of treatments during diesel bioremediation over time.

**Fig. 5.**
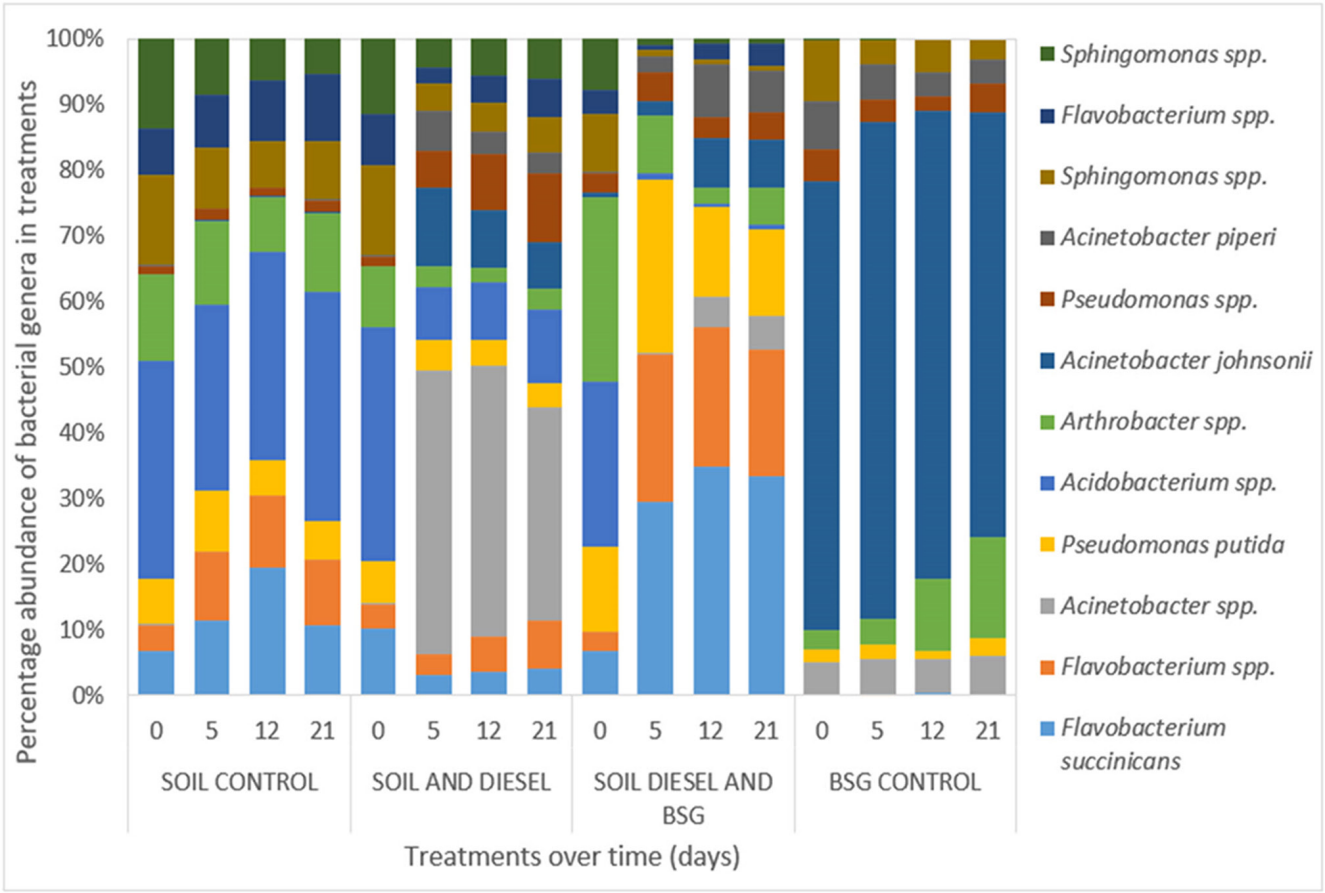
Bacterial community profile showing percentage abundance of bacterial populations during diesel bioremediation.

Results monitoring the bacterial community changes and dynamics during the transformation of aliphatic and aromatic hydrocarbons in the diesel contaminated soil during bioremediation were profiled in terms of the relative abundance (Fig. 4) and percentage abundance (Fig. 5) of the 12 most dominant bacterial populations in the treatments. Aliquots from the same community genomic DNA samples, with and without BSG, used in the qPCR assay for the three catabolic genes in this study were used for this analysis for comparability with each other and with the control soil alone and BSG alone samples. DNA yield from contaminated samples (µg) were all high and the purity based on absorbance at 260 and 280 nm using a Nanodrop (ND-2000; Thermo Fisher Scientific) were between 1.8 and 2.0 as expected. Diesel contamination did not appear to impact genomic DNA recovery from the soil.

The results in Fig. 4 show that the treatment amended with BSG had the highest relative abundance of bacterial populations. These results are consistent with those from culture-based methods in which treatments amended with BSG supported the highest heterotrophic and hydrocarbonoclastic bacterial populations. Also, the bacterial populations on day 0 were similar for all treatments except that of the grain control. The grain control, however, did not continually sustain microbial growth on its own.

As shown in Fig. 5, we observed in the soil alone control treatment (S) that the percentage abundance of *Flavobacterium succinicans* increased from around 7 % on day 0 to 20 % on day 12. Thereafter, it reduced to 11 % on day 21. Culturable hydrocarbon degrading bacteria were first observed on day 7 in this treatment. Apart from these changes, no significant shifts in the percentage composition of other bacterial genera in this treatment were observed.

In the BSG alone control treatment, the percentage abundance of *Arthrobacter* spp. increased from 2 to 15 % from the start of the experiment up till day 21 (Fig. 5). Slight decreases in the abundance of *Pseudomonas* spp., *Acinetobacter piperi* and *Sphingomonas* spp. were also observed. As with the control soil alone treatment, there were no significant changes in the percentage composition of other bacterial populations in this treatment, with *Acinetobacter johnsoni* being the most abundant species in this treatment throughout the experiment.

In contrast, the percentage composition of bacterial populations in the diesel contaminated soil treatments changed extensively. A rapid shift in bacterial community composition was observed in the unamended soil and diesel treatment after day 0. The population profile revealed three *Acinetobacter* species in the treatment by day 5, namely: *Acinetobacter* spp. 45%, *Acinetobacter johnsonii* 12% and *Acinetobacter piperi* 5 % while *Acidobacterium* spp. were reduced. Changes in species abundance were observed hereafter though no more shifts in community composition were seen. After day 5 the three *Acinetobacter* species gradually reduced while two *Flavobacterium* species increased. *Acinetobacter* spp. were the most abundant species in this treatment though it reduced from 45% to 41% and to 32 % on days 5, 12 and 21, respectively. *Acinetobacter johnsonii* decreased from 12% to 8% and to 5 % on days 5, 12 and 21 respectively and *Acinetobacter piperi* decreased from 5% to 3% and to 2.5 % on days 5, 12 and 21. *Flavobacterium* spp., however, increased from 3% to 5% and finally to 9 % on days 5, 12 and 21 while *Flavobacterium succinicans* increased from 2% to 3% and to 5 % on the same days. *Pseudomonas* spp. also increased from 5% to 8% and finally to 10 %.

Addition of BSG to the contaminated soil on day 0 may have contributed to the increased percentage abundance of *Arthrobacter* spp. and *Pseudomonas putida* observed in the amended treatment (S+D+G) compared to the unamended soil and diesel (S+D), and soil control (S) treatments (Fig. 5). A rapid shift in community composition was observed in this treatment by day 5, resulting in the increased abundance of *Flavobacterium succinicans *, *Flavobacterium* spp. and *Pseudomonas putida *. Thereafter, this treatment sustained these three species as the most abundant throughout the experiment. At the same time, *Arthrobacter* spp. declined and *Acidobacterium* spp. were almost eliminated. As with the unamended treatment, a reduction in the abundance of *Acidobacterium* spp. following contamination was also observed in the amended treatment. *Acinetobacter piperi* was also observed in this treatment after day 0. After day 5, a second shift in bacterial community composition was observed in the amended treatment during which *Acinetobacter* spp. were seen and the abundance of two *Acinetobacter* species increased. *Acinetobacter johnsonii* increased from 2% to 8% and to 9 % and *Acinetobacter piperi* increased from 4% to 9% and to 8 % on days 5, 12 and 21 respectively. At the same time, however, the abundance of *Pseudomonas putida* decreased from 28 to 14 %.

## Discussion

An enhancement of the bioremediation process by stimulating with BSG was evident by the significant reduction in percentage TPH and high biodegradation rates observed. Similarly, Agarry and Latinwo [36] reported a high rate of 79 % TPH reduction after 28 days when using brewery spent effluent for the bioremediation of a 10 % (w/w) diesel contaminated soil. The potential of BSG to enhance the bioremediation process was also shown by the significant increase in both heterotrophic and hydrocarbonoclastic bacterial populations, along with a negative correlation between petroleum hydrocarbon degradation and bacterial CFUs [11, 59]. The CFUs observed in the soil only control reveal that the soil contains indigenous hydrocarbon degraders which began utilising diesel as their only carbon source after a lag phase of about 1 week. Similarly, in their study, Alisi *et al*. [60] reported that in soils without prior hydrocarbon contamination, a 2 day lag phase was observed before CO_2_ evolution and 6 days were required for the soil microbial community to become active.

The initial rapid TPH reduction observed in this study was contributed to by all the aliphatic TPH (C10–C28) hydrocarbon fractions. However, although the C10, C12 and C28 aliphatic fractions in the amended treatment were completely degraded, the C12 fraction, having a lower molecular weight despite its higher initial concentration, degraded faster than the C28 fraction. Thus, it is likely that the most labile and low molecular weight fractions were metabolized in the initial rapid phase while the more resistant and higher molecular weight fractions were degraded in a later, second phase [61–63]. The initial rapid degradation in both the natural attenuation and amended treatments may also have resulted from autochthonous hydrocarbon degraders being present in the soil [11]. Although the soil was pristine, hydrocarbonoclastic microbes are known to be ubiquitous, and following contamination it has been observed that microbial communities in pristine soils adapt well to contaminants, resulting in rapid degradation [64–66].

The degradation pathways of aliphatic hydrocarbons show that, depending on the position of the methyl group initially attacked by the oxygenase enzyme, alkanes may be oxidized to either primary or secondary alcohols [58]. Further oxidation of primary alcohols produce aldehydes and fatty acids while secondary alcohols produce ketones and esters [5]. The degradation pathways of aromatic hydrocarbons, however, indicate that they are initially oxidized to catechol (benzene-1, 2-diol) [15, 67]. Thereafter, cleavage of the benzene ring occurs in either of two routes: the *ortho*-cleavage pathway, which involves cleavage between carbons 1 and 2 catalysed by catechol-1, 2-dioxygenase to produce ketones and esters, and the *meta*-cleavage pathway, which involves cleavage between carbons 2 and 3 by catechol-2, 3-dioxygenase to produce aldehydes and carboxylic acids [57, 68]. From the elucidated pathways, and as observed during this study, ketones and aldehydes are key distinguishing metabolites in determining the catabolic pathways utilized by the bacterial community. Aldehydes are the distinguishing metabolites for the terminal oxidation pathway of aliphatics and *meta*-oxidation pathway of aromatics [58, 68, 69]. Ketones, on the other hand, distinguish the activity of the subterminal oxidation pathway of aliphatics and the *ortho*-oxidation pathway of aromatics [5, 58].

According to Tsugawa *et al*. [70], to deduce the metabolic activity of microbes associated with hydrocarbon biodegradation, an analysis of metabolites in the biological samples is required. Thus, to deduce the metabolic pathways in this study, we identified ketones and aldehydes from the literature [5] as the signature metabolites that differentiate between the terminal and subterminal pathways in aliphatic hydrocarbon degradation catalysed by alkane monoxygenase and the *ortho-* and *meta*-degradation pathways of aromatic hydrocarbon degradation catalysed by catechol-1, 2-dioxygenase and catechol-2, 3-dioxygenase respectively [63, 71]. Aliphatic hydrocarbons may have been degraded mainly via the subterminal oxidative pathway as suggested by the presence of aliphatic ketones and esters, in both the natural attenuation and amended treatments, throughout the biodegradation process (Table 4). However, the presence of aliphatic fatty acids (carboxylic acids), though aliphatic aldehydes were not detected, suggests that a rapid oxidation of aldehydes to carboxylic acids may have occurred, particularly in the BSG-amended treatments. The carboxylic acids may then have been oxidized via the β-oxidation pathway to the TCA cycle.

For aromatic hydrocarbon degradation, however, aromatic ketones were present throughout the degradation, indicating that degradation was mostly via the *ortho*-cleavage pathway catalysed by catechol 1, 2-dioxygenase [71]. Aromatic aldehydes, which are the key differentiating metabolites of the *meta*-cleavage pathway catalysed by catechol-2, 3-dioxygenase [63], were only present at the start of the degradation in the natural attenuation treatment. However, the amended treatment with BSG supported its presence from day 5 of the degradation process and throughout the experiment (Table 4). Thus, the metabolites present at different times during the bioremediation process in this study have shown that the breakdown of diesel was mostly via the subterminal oxidation pathway for the aliphatic hydrocarbon content of diesel and via the *ortho*-oxidation pathway for the aromatic hydrocarbon content of diesel.

The amendment of diesel contaminated soil with BSG appeared to enhance metabolism via these two pathways, while also sustaining metabolism via the *meta*-cleavage pathway for aromatic hydrocarbon degradation and speeding up the metabolism of carboxylic acids via the β-oxidation pathway for aliphatic hydrocarbons in terms of synthesis or appearance of metabolic substrates and catabolism of metabolic products. Thus, diesel degradation in this study fits the elucidated pathways, is consistent with the literature [27, 63, 72] and provides evidence that the amendment of contaminated soil with BSG enhances the oxidative breakdown of hydrocarbons.

Knowledge of the degradation pathway, as determined by the metabolites present, provided information on the degradation genes to be assayed and related the degradation pathways to their associated degradation genes. DNA sequencing targeting the 16S rRNA gene amplicon, on the other hand, provided information regarding the community bacterial populations associated with the biodegradation, thus correlating these degradation genes with the bacterial population harbouring them and further confirming the bioremediation potential of BSG. Among the three associated genes of the degradation pathways in this study, the *alkB* gene, which is key in the aerobic biodegradation of aliphatics [22, 51, 73, 74], had the highest percentage increase from the start to the end of the bioremediation, especially in the amended treatment. This, interestingly, tallies with the presence of the aliphatic ketone metabolites throughout the entire biodegradation experiment. Similar increases in *alkB* gene copy numbers have been recorded during the bioremediation of a 1 % diesel and engine oil contaminated soil using plant residues [11].

The combination of microbiome analysis with GC-MS evaluation of metabolites adopted in this study is useful in elucidating the bacterial community dynamics during diesel bioremediation. The results tally with that of the elucidated degradation pathways such that the abundance of aromatic ketone metabolites from the start of the experiment (Table 4) was reflected in the abundance of the *catA* gene, which was the only gene having a significantly higher copy number from the start of the degradation. This coincided with the rapid hydrocarbon biodegradation observed at the onset of the degradation process. Since the *catA* gene encodes the catechol-1, 2-dioxygenase enzyme, responsible for metabolizing aromatic hydrocarbons through the *ortho*-cleavage pathway and having ketone metabolites, its activity may have contributed to the statistically significant reduction of hydrocarbons in the treatments amended with BSG. A similar rapid initial degradation phase was also observed in the study of Ros *et al*. [62].

*Pseudomonas putida,* which was abundantly present in the treatment amended with BSG from the start of the experiment and sustained throughout the experiment in this treatment (Fig. 5), declined after day 5. Similarly, the *catA* gene copy numbers (Fig. 3b) also declined by day 5. *P. putida* is known to metabolize both aliphatic and aromatic hydrocarbons [75] and has the *catA* gene that metabolizes aromatic hydrocarbons via the *ortho*-cleavage pathway catalysed by catechol-1, 2-dioxygenase [76, 77]. *P. putida* was also present in the treatment without BSG, in which hydrocarbons were also reduced during the initial rapid biodegradation. Nevertheless, the abundance of *P. putida* was greater in the amended treatment than in the unamended treatment. This suggests that *P. putida* may have been actively involved in the metabolism of aromatics catalysed by catechol-1, 2-dioxygenase to produce ketones at the start of the experiment and contributed to the rapid initial degradation observed. Further experiments showing the breakdown pattern of aromatic hydrocarbons revealed that aromatic hydrocarbons were mostly degraded at the initial degradation phase [53].

A major shift in microbial community in favour of *Acinetobacter* species was observed following the introduction of diesel to the natural attenuation treatment. *Acinetobacter* is a known hydrocarbon degrading genus, identified as having the *xylE* gene encoding catechol-2, 3-dioxygenase that catalyses the degradation of aromatic hydrocarbons via aldehyde metabolites [18, 78]. This gene has also been detected in *Pseudomonas* spp. [18, 78]. *Acinetobacter* was identified in the unamended soil and diesel treatment immediately after the start of the degradation process. It is not surprising, then, that aromatic aldehydes were identified in this treatment at the start of the degradation. However, as the abundance of *Acinetobacter johsonii* and *Acinetobacter piperi* decreased in the unamended treatment after day 5, both species increased in abundance until the end of the experiment in the amended treatment. This was also reflected by the presence of aromatic aldehydes in the amended treatment as from day 5 until the end of the experiment and although *Acinetobacter* spp. persisted in the unamended treatment, aldehydes were no longer seen in the unamended treatment.

Community profiling has shown that the genera *Acinetobacter* and *Pseudomonas,* among others, may be responsible for the metabolism of hydrocarbons in this study. These genera were also observed to be present in BSG. *Flavobacterium *, however, was seen to be the most abundant genus in the amended treatment. Nevertheless, it is likely that a synergy of microbes is necessary for the complete degradation of hydrocarbon contaminants rather than a single species. This tallies with previous studies showing that species of *Acinetobacter, Arthrobacter, Flavobacterium, Nocardia, Pseudomonas* and *Vibrio* are associated with petroleum hydrocarbon degradation [5, 31]. Since *Flavobacterium* species have been identified as degraders of hydrocarbons [5, 11, 79], their increase in the soil alone control treatment may be the reason behind the presence of hydrocarbon utilising bacterial CFUs in this treatment as from day 7. *Acidobacterium* spp., however, reduced in abundance following hydrocarbon contamination. This is interesting to note as they are ubiquitous and mostly found in soils but not known hydrocarbon degraders [80].

The adoption of molecular techniques in this study provided a culture independent approach in the elucidation the bacterial population dynamics during the biodegradation of diesel contaminated soil amended with BSG. Changes in the abundance and an enrichment of autochthonous aerobic hydrocarbon degraders resulting in shifts in the bacterial population in their favour were evident. Also, community profiling of the BSG alone control treatment showed that the grain supports its own microflora, most of which are known hydrocarbon degraders. This supports the potential of supplementation with BSG as not just a biostimulation treatment but also bioaugmentation.

## Conclusion

The findings of this study, including microbiological, metabolite and genetic analysis to assess bioremediation, provide a more informed understanding of the process. They also elucidate the correlation between these parameters in monitoring bioremediation to allow for the design of more effective interventions. The study has demonstrated that the amendment of diesel-contaminated soil with BSG enhanced biodegradation under controlled conditions reflecting temperate environments. BSG contains intrinsic hydrocarbon degrading species including *Pseudomonas putida* and *Acinetobacter piperi* and promoted increased gene copy numbers of the *alkB*, *catA* and *xylE* genes. Organic by-products such as BSG can thus provide a valuable contribution to bioremediation as well as reducing potential landfill disposal, making it an environmentally viable option.
